# Progress and challenges in development of animal models for dengue virus infection

**DOI:** 10.1080/22221751.2024.2404159

**Published:** 2024-09-23

**Authors:** Wang Yuya, Yang Yuansong, Liu Susu, Ling Chen, Wu Yong, Wang Yining, Wang YouChun, Fan Changfa

**Affiliations:** a Institute for Laboratory Animal Resources, National Institutes for Food and Drug Control (NIFDC), Beijing, People’s Republic of China; b College of Life Science school, Northwest University, Provincial Key Laboratory of Biotechnology of Shaanxi Province, Xi’an, People’s Republic of China; c Institute of Medical Biology, Chinese Academy of Medical Sciences and Peking Union Medical College, Kunming, People’s Republic of China

**Keywords:** Dengue virus, potential receptor, animal models, pathogenesis, ADE and vaccine

## Abstract

The severity of the dengue epidemic is on the rise, with its geographic range had expanded to southern Europe by 2024. In this August, the WHO updated the pathogens that could spark the next pandemic, dengue virus was on the list. Vaccines and drugs serve as powerful tools for both preventing dengue infections and treating patients. Animal models play a pivotal role in vaccine development and drug screening. Available potential susceptible animals, including non-human primates, rodents, pigs, and tree shrews, have been extensively explored to establish animal models of dengue disease. Despite significant advancements, there are still notable limitations. Different animal models exhibit distinct constraining factors such as viraemia, host susceptibility, immune function of the host, clinical symptoms, ADE (antibody-dependent enhancement) phenomena, cytokine storm response to various serotypes and strain variations. Furthermore, despite extensive research on the dengue virus receptor in recent years, genetically modified animal models immunocompetent harbouring dengue virus susceptibility receptors have not yet been available. This work reviewed the research progress of dengue virus receptors and dengue animal models, suggesting that the development of genetically modified murine models expressing dengue virus functional receptors may hold a promise for future dengue disease research, especially for its vaccine development.

## Introduction

The Dengue outbreak, caused by the dengue virus (DENV), is considered one of the most significant arthropod-borne viral infections affecting human populations in various tropical and subtropical regions [1]. In the updated pathogens that could spark the next pandemic issued by WHO in August 2024, dengue virus was back on the list [2]. Currently, approximately one third of the global population is at risk of DENV infection, with an estimated 400 million people becoming infected each year [3,4]. The ongoing spread of the dengue epidemic globally can be attributed to abrupt climate changes, rapid unplanned urbanization and construction, high population density, and ineffective vector control strategies [3]. It is imperative for the scientific community to increase its focus on addressing this persistent dengue epidemic. Particularly in 2023, there have been over 5 million reported infections globally, including 5000 deaths, nearing a historic peak. Further, new regions across the world are being affected, including southern Europe [5]. Furthermore, as of April 2nd in 2024, it has been reported that the dengue virus has resulted in over 3,000,000 infections and 80 deaths in Brazil and more than 180,000 infections and 129 deaths in Argentina (WHO Dengue and severe dengue: https://www.who.int/news-room/fact-sheets/detail/dengue-and-severe-dengue; Dengue: 21 estados e o DF apresentam queda ou estabilidade na incidência da doença：https://www.gov.br/).

**Figure 1.**
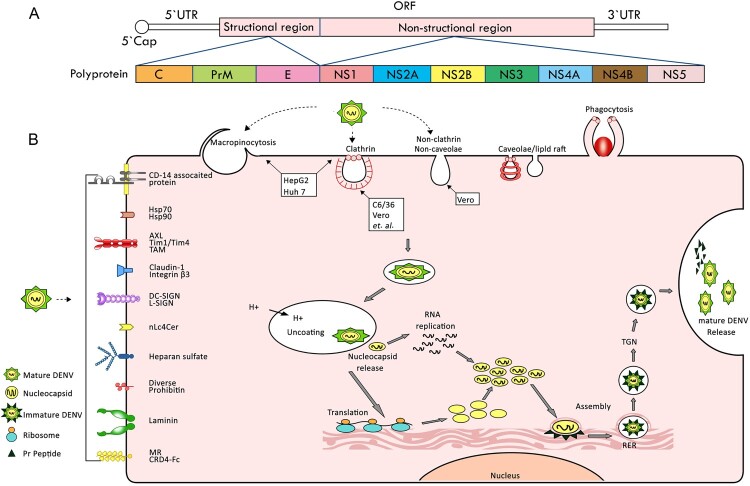
(A) Diagram of the DENV genome. (B) Dengue receptor, dengue entry and life cycle.

DENV is a single positive stranded RNA virus with a genome length of approximately 10.7 kb, classified within the family Flaviviridae and the genus Flavivirus [6]. The DENV genome encodes three structural proteins – capsid (C), membrane (M) and envelope (E) proteins, as well as seven nonstructural proteins NS1, NS2A, NS2B, NS3, NS4A, NS4B and NS5 [4,7] (Figure 1(A)). There are four common serotypes of dengue infection, namely DENV-1 to DENV-4 [3]. A new serotype, DENV-5, was discovered in Malaysia in 2007 [4,8]. Infection with the same serotype may confer lifelong immunity; however, only short-term immunity is maintained against hetero-infection and may even lead to severe dengue due to antibody dependent enhancement (ADE) [3]. Individuals infected with dengue virus can range from being asymptomatic or having mild dengue, neurological complications to developing severe conditions such as Dengue hemorrhagic fever (DHF) and Dengue shock syndrome (DSS), which can be life-threatening [9,10]. Severe dengue fever is characterized by plasma leakage, bleeding tendency, organ failure, shock, and occasionally death [9,10].

Animal models have proven to be indispensable tools for dengue epidemic prevention, with significant implications in understanding pathogenesis, assessing vaccines, identifying drugs, and developing treatments [11]. Scientists have been striving to establish the models with DHF and DSS akin to dengue infection in humans, a long-standing and challenging pursuit. The dengue virus receptor is an imperative molecule that mediates host infection and essential for establishing an ideal animal model. This review presents an overview of potential receptors for DENV, recent mammalian models utilized in DENV studies, encompassing NHPs, pig and tree shrew models, as well as the obstacles encountered by mouse models.

## Receptors for DENV – important but far from conclusive

DENV is internalized into acidified endosomes upon cellular entry, where the nuclear capsid is released into the cytoplasm [7]. Subsequently, the RNA genome undergoes uncoating and is then transported to the endoplasmic reticulum for translation. Then viral genome replication, assembly and release, followed by dissemination and infection of neighbouring cells [4] (Figure 1(B)).

DENV enters cells *via* endocytosis mediated by cell surface molecular receptors including AXL [12], CD14-associated protein [12], Claudin-1 [12], C-type lectin domain family 5 member A (CLEC5A) [6], CRD4-7-Fc [13], DC-SIGN/L-SIGN [14], Diverse [12], FcγR [15,16], GRP78 [12], Heparan sulphate [17], Laminin receptor [12], HSPGs[18], HSP70/90 [12,19], Integrin β3 [20], Mannose receptor (MR, CD206) [13], nLc4Cer [21], prohibitin [12,22], Tim-1/Tim-4 and TAM family [23] (Figure 1 and Table 1). The specificity of the receptor proposes with some controversy. Notably, almost these receptor candidates but CLEC5A, FcγR and Integrin β3, have demonstrated DENV susceptibility *in vitro* without any available evidence *in vivo* (Table 1). It is reported that CLEC5A/TLR2 mediated neutrophil extracellular traps formation and inflammatory reactions enhancement could increase lethality induced by DENV in STAT1-deficient mice [6,24]. FcγRIIIa has demonstrated to act on splenic macrophages to mediate the pathogenesis of DENV antibodies, resulting in inflammatory sequelae and mortality [15,16]. And a novel mechanism in which interaction between integrin β3 and domain III of E protein (EDIII) is involved in DENV entry in AG129 mice contributes to anti-DENV drug design [20]. The receptor knock-in mouse model of SARS-CoV-2 [25,26], EV-A71 [27,28] and other viruses has exemplified that viral receptors shed light on susceptibility, transmission, pathogenesis, drug development and treatment, as well as vaccine advancements. Unfortunately, despite years of dedicated research, numerous receptor candidates have been identified, yet the development of a receptor-based animal model for dengue-susceptible mice remain pending. It is worth noting that the ongoing controversy about the specificity of DENV receptors presents a significant challenge in building the susceptible genetically modified murine models and may even lead to strategic errors.

**Table 1.** Possible receptors and proteases for dengue entry.

Cellular receptors	Properties	Serotype	In vivo supporting data available or not	Refs
AXL	TAM family with the high-affinity ligand growth arrest-specific protein 6	DENV-2/3	No AXL expressing mouse model. Cellular evidence available	[12]
CD14-associated protein	Protein associated with LPS receptor	DENV-2	No CD14-associated protein expressing mouse model; Cellular evidence available	[12]
Claudin-1	Major structural component of tight junction, dengue entry	DENV-2	No Claudin-1 expressing mouse model; Cellular evidence available	[12]
CLEC5A	C-type lectin domain family 5 member A, pattern recognition receptor	DENV-2	No mouse model expressing CLEC5A; In vivo supporting data available in STAT1-deficient mice; Cellular evidence available	[6,24]
CRD4-7-Fc	A recombinant MR fusion protein	DENV-1/2/3/4	No mouse model expressing CRD4-7-Fc; Cellular evidence available	[12]
DC-SIGN/L-SIGN	DC-specific ICAM 3-grabbing nonintegrin (DC-SIGN, CD209)/ liver/lymph node-specific ICAM 3-grabbing integrin (L-SIGN)	DENV-1/2/3/4	No mouse model expressing DC-SIGN/L-SIGN; Cellular evidence available	[14]
Diverse	Diverse protein of mosquito tissues	DENV-2/3/4	No mouse model expressing Diverse; Cellular evidence available	[12]
FcγR	An alternative receptor, antibody-virus complexes, ADE of infection	DENV-2	No mouse model expressing FcγR; In vivo supporting data available in STAT1-deficient mice in IFNAR1−/−/FcγR KO and IFNAR1−/−/FcγR humanized mice; Cellular evidence available	[15,16]
GRP78	Receptor elements in dengue entry process	DENV-2	No mouse model expressing GRP78; Cellular evidence available	[12]
Heparan sulphate	Nonspecific receptor for dengue attachment, expressed in almost all cell types	DENV-1/2/3/4	No mouse model expressing Heparan sulphate; Cellular evidence available	[17]
Laminin receptor	High-affinity laminin receptor	DENV-1/2/3	No mouse model expressing Laminin receptor; Cellular evidence available	[12]
HSPGs	Heparan sulphate proteoglycans,	DENV-1/2/3/4	No mouse model expressing HSPGs; Cellular evidence available	[18]
HSP70/HSP90	Dengue entry receptor, CD-14-independent cell surface functional receptors	DENV-2	No mouse model expressing HSP70/HSP90; Cellular evidence available	[12,19]
Integrin β3	Dengue entry receptor	DENV-2	No mouse model expressing Integrin β3; In vivo supporting data available; Cellular evidence available	[20]
Mannose receptor (MR, CD206)	A highly effective endocytic receptor with a broad binding specificity	DENV-1/2/3/4	No mouse model expressing MR; Cellular evidence available	[13]
nLc4Cer	Galβ1-4GlcNAcβ1-3Galβ1-4Glcβ1-1’Cer; Glycosphingolipid	DENV-1/2/3/4	No mouse model expressing nLc4Cer; Cellular evidence available	[21]
Prohibitin	Specific for DENV-2 entry in C6/36 cells	DENV-2	No mouse model expressing prohibitin; Cellular evidence available	[12,22]
Tim-1/Tim-4, TAM family	PtdSer-dependent phagocytic removal of apoptotic cells, serve as dengue entry	DENV-2/3	No mouse model expressing Tim-1/Tim-4; Cellular evidence available	[12,23]

## Animal models

### NHP models

NHPs share close physiological and genetic similarities with humans, making them valuable animal models for studying infection and vaccine development (Table 2). Preliminary research in vaccine development typically includes assessments of safety, viraemia, clinical signs, antigenemia, immunogenicity, and antibody efficacy among other parameters [29]. Infection with 10^4^∼10^6^ PFU of DENV is considered to mimic the inoculum in mosquito bites [11]. Rhesus monkeys are the first models used to study dengue etiology through subcutaneous and intravenous inoculation of purified blood from dengue patients. The clinical manifestations rarely mirror those observed in human patients and low viremia limits it application in vaccine development [11,29–33]. Bonnet macaques present high viremia [34,35] and chimpanzees exhibit low viremia [11]. Compared to another NHPs, Marmosets display clinical symptoms such as fever, leukopenia, thrombocytopenia, hematuria and renal injury, exhibiting some features of severe dengue [11,34]. In primary infection, cynomolgus macaques experience a moderate viremia for 2∼4 days (peak average: DENV-1 strain 02–17 10^2.8 copies/mL, DENV-2 strain DHF0663 10^5.2 copies/mL, DENV-3 strain DSS1403 10^2.8 copies/mL) [36-38], and marmosets experience a high viremia for 4 ∼ 8 days (peak average: DENV-1 02–17 10^6.6 copies/mL, DENV-2 DHF0663 10^7.3 copies/mL, DENV-3 DSS1403 10^5.6 copies/mL) [34,39]. Similarly, in secondary DENV infection, cynomolgus macaques develop a 3 d’s viremia duration (peak average: strain DENV-2 DHF0663 10^6.2 copies/mL, DENV-3 strain DSS1403 10^2.9 copies/mL) [36-38] and marmosets develop a 7 ∼ 10 d’s viremia duration (peak average: DENV-2 strain DHF0663 10^6.4 copies/mL, DENV-3 strain DSS1403 10^6.8 copies/mL) [34,39]. Marmoset infections last a higher and longer viremia than cynomolgus macaques. Besides, marmosets cause high levels of viremia in wild isolates challenge group and low to undetectable levels of viremia in candidate vaccine group in an evaluation study of one candidate vaccine [40].

**Table 2.** Dengue virus infection models in NHPs.

Animal species	Serotype	Clinical signs	Limitations	Primary application	Refs
Rhesus monkey	DENV-1/2/3/4	Low blood viremia, T-cell response, nAbs production, PLT decrease, hemorrhage, thrombocytopenia and neutropenia	Low viremia, No manifestations of DHF/DSS, vascular leaks, high cost	DENV infection course; Dengue pathogenesis; Vaccine evaluation; ADE model	[11,29–33]
Marmoset	DENV-1/2/3/4	High blood viremia, protective antibody production, serotype cross-reactive nAb responses, T and NKT cell response, kidney injury, fever, thrombocytopenia	No discernible clinical signs	High blood viremia; Primary and secondary dengue infection; Immunogenicity and vaccine evaluation; Dengue pathogenesis	[29,34,39,40]
Cynomolgus macaques	DENV-1/2/3/4	IgM and IgG production	No discernible clinical signs	DENV infection; Vaccine efficacy;	[29,36–38,41,42]
Bonnet macaque	DENV-4	High blood viremia, antibody production	No discernible clinical signs	High blood viremia;	[34,35]
Chimpanzee	DENV-1/2/3/4	Low blood viremia, leukopenia, nAbs production	No discernible clinical signs; Low blood viremia; High cost	Immune response	[11]

The investigation of immune response triggered by DENV infection in NHPs has garnered considerable attention. Specifically, the evaluation of IgG and IgM, pivotal constituents of humoral immune response responsible for virus neutralization and clearance, holds significant implications for assessing infection progression, immune response intensity, and disease severity [29]. In primary dengue virus infection, cynomolgus macaques produce dengue-specific IgM within 1 ∼ 3 days [41], whereas marmosets do so within 3 ∼ 5 days [34,39]. For secondary heterologous infections, cynomolgus macaques IgM 5 days later [41,42], while marmosets do so 7 days later [34,39]. Subsequently, the gradual decrease in IgM levels serves as a crucial indicator of early-stage virus infection. Regarding IgG, dengue-specific IgG is detected in cynomolgus macaques 7 days post initial infection [41] and in marmosets 5 days thereafter [34,39]. Both cynomolgus macaques and marmosets exhibit earlier detection of IgG by 3 days during secondary infections [29]. Cynomolgus macaques produce neutralizing IgG antibodies 28 days post-infection, while marmosets do so at day 14 [29]. High levels of IgG can persist for months or even a lifetime [29,42]. It is noteworthy that the production of IgG antibodies varies across different serotypes of DENV infection. Neutralizing IgG antibodies can confer lifelong neutralization against the same type of virus; however, cross-reactive neutralization ability against heterotypic viruses diminishes over time and may lead to ADE, exacerbating infection and disease progression. This immune response pattern mirrors the antibody response observed in human dengue virus infection [42].

NHP animal models are often highly anticipated, but the NHP models of DENV have failed to meet expectations by not replicating the significant clinical symptoms of DENV infection. Marmosets could be better served as an NHP model that demonstrates the protective capacity of candidate dengue vaccines. However, its viremia levels and duration during dengue infection are inferior to be observed in humans. In addition, obtaining genetically modified NHP expressing human dengue virus receptors is difficult, including technical obstacles as well as ethical concerns.

### Mouse models

#### Immunocompetent model for DENV

Immunocompetent mice of DENV infection have obtained significant advancements (Table 3). For BALB/c mice, non-mouse-adapted DENV-2 infection causes serum ALT and AST increase, a Th1 bias immune response and low viremia [43], neuro-adapted DENV-2 develops encephalitis [44,45]. In C57BL/6 mice, DENV-2 (strain 16681) lead to hyperviremia, systemic hemorrhage, platelet decrease, liver enzyme rise and a Th2 bias immune response [45,46]. Furthermore, infection of C57BL/6 mice with non-mouse-adapted DENV-1 (Puerto Rico/94) and DENV-2 (Tonga/74) resulted in low platelet count, internal hemorrhage and elevated liver enzyme [43,47]. Atypical anti-DENV CD8 + T lymphocytes were found to play an important role in severe dengue fever [48]. Sulforaphane suppresses dengue virus replication and prolongs survival time of the DENV infected ICR mice [49]. That DENV NS1 might associate with local skin hemorrhage has demonstrated in C3H/HeN mice [50].

**Table 3.** Immunocompetent mouse model of dengue virus infection.

Mouse model	Serotype	Clinical signs	Limitations	Primary application	Refs
BALB/c	DENV-1/2/3/4	Fever, low blood viremia, neurologic signs, liver injury, kidney damage, hemorrhage, lethality, Th2 type bias	Lack human clinical signs; low blood viremia; No DENV specific antibody and T-cell response	Lethality for DENV-3; Immunopathogenesis; Cytokines storm Th2 type bias; nAbs	[43-45]
C57BL/6	DENV-1/2/3	Fever, low blood viremia, neurologic signs, systemic hemorrhage, vascular leakage, thrombocytopenia, liver damage, macrophage infiltration, Th1 type bias, lethality	Lack discernible clinical signs; low blood viremia; immune response	Thrombocytopenia; Severe dengue; Immunopathogenesis; Susceptibility than BALB/c; Cytokines storm Th1 type bias; neutralizing antibody	[45-48]
ICR	DENV-1/2	blood viremia; neutralizing, lethality	lack human clinical signs	Immunopathogenesis	[49]
C3H/HeN	DENV-2	blood viremia, thrombocytopenia, bleeding	Lack human clinical signs	Immunopathogenesis	[50]

Inoculation of immunecompetent BALB/c, C57BL/6, ICR and C3H/HeN mice with DENV result in viremia, thrombocytopenia, production of antiplatelet antibodies, neurological symptoms, and mortality. However, these manifestations are still insufficient to fully replicate the clinical features of human beings.

#### Immunocompromised mouse models for DENV

The endogenous interferons (IFNs) production is regulated by IFN regulatory factors (IRF) 1/3/5/7, and IFN receptors and STAT1/2 are pivotal molecules in the response stage, both of which are implicated in dengue virus infection and inflammatory response [51]. Consequently, a series of infectious mouse models have been developed to assess DF/severe DF through IFN signalling pathway and inflammatory response (Table 4).

**Table 4.** Immunocompromised mouse model of dengue virus infection.

Mouse model	Serotype	Clinical signs	Primary application	Limitations	Refs
AG129	DENV-1/2/3/4	High viremia, paralysis, lethal, vascular leakage, spleen/liver damage, thrombocytopenia, nAbs production	Neurologic signs, Mild and severe dengue model; Lethal DENV infection model; Drug testing and pathogenesis studies; Immunogenicity and vaccine evaluation	Lack discernible clinical signs; Natural infection; Thrombocytopenia; DENV infectivity depends on highly mouse-adapted DENV strains; Age-dependent disease severity	[11,53–60]
LysM-Cre + IFNARfl/fl	DENV-2/3	Vascular leakage, liver injury, thrombocytopenia	Better immune response than AG129; Vaccine candidate; ADE model	Pan-serotype	[61]
STAT1-/-STAT2-/-	DENV-2	Viremia, lethal outcome	Anti-DENV defense mechanism	Natural infection	[62]
IRF3-/-IRF5-/-IRF7-/-(TKO)	DENV-2	viremia	Antiviral role of IRF-1 by inducing IFN responses against DENV infection	Natural infection	[63]
hTNF++	DENV-2	Neurological symptoms; no viremia and T-cell responses	DENV encephalitis	Natural infection	[43,64]
CCR5-/-	DENV-2	Resistant to lethal infection	DENV replication	Natural infection	[65]

Currently, AG129 mice (type I and II IFN receptor double-knockout mice) represent the preferred model for lethal dengue fever research [43,52] and vaccine evaluation [53]. Notably, different serotypes, strains and inoculation routes can all elicit the changes of clinical manifestations of DENV infection in the AG129 mouse model. Studies have demonstrated that clinical isolates of both DENV-1 (strain WP 74) and DENV-2 can elicit severe manifestations of DF in AG129 mouse models [11,54]. The first report for AG129 infected with brain-adapted DENV-2 strain New Guinea C (NGC) developed clinical signs culminating in death by day 12 post-inoculation, with one drawback of severe neurological signs [53]. Subsequently, the researchers infected mosquito cells and AG129 mice with both NGC and PL046 in alternating fashion, resulting in a novel virus strain DENV-2 D2S10 generation. This strain can infect AG129 mice and induce vascular permeability enhancement leading to a fatal disseminated disease without neurological manifestations, thus better modelling human DHF/DSS. Then again, new non-mouse-adapted DENV-2 S2-21 and D2Y98P strains emerge [55,56]. These newly identified strains suggest that dengue virus undergoes lots of selection pressure after infections in the animal models. The DENV-2 16681 or PDK-53 strains have been associated with paralysis, death, and pronounced neurological symptoms [57]. Additionally, the non-adapted DENV-3 C0360/94 strain infection can result in fatal outcomes characterized by liver and spleen damage, elevated cytokines, vascular leakage, thrombocytopenia, and leukopenia but without neurological manifestations [58]. Conversely, infection with DENV-3 D83-144 strain can lead to only mild dengue fever symptoms [59]. Furthermore, intraperitoneal infection with DENV-4 TVP-376 strain results in mortality within 5 days for AG129 mice along with widespread viral dissemination across various tissues [60]. CDIIc/LysM-Cre ^+ ^IFNAR^fl/fl^ mice (overexpression IFNAR in IFNAR^-/-^ mice) exhibit a heightened immune response to DENV infection compared to IFNAR^-/-^ mice, as the tool of potential vaccine candidates [61]. STAT1^-/-^STAT2^-/-^mice succumb in early infection [62], indicating the resistance of the STAT1/2-mediated IFN signalling pathway to DENV-2 (strains S221, 16681, D2S10) infection [62]. IRF3^-/-^IRF5^-/-^IRF7^-/-^ (TKO) mice also elucidate the antiviral defense mechanism mediated by IFN signalling pathway in severe DF [63]. hTNF^+++^ C5BL/6J mice develop neurological symptoms following DENV-2 (strain PL046) infection [43,64], while CCR5^-/-^ mice demonstrated resistance to fatal following DENV-2 (strain P23085) infection [65]. In conclusion, despite the robust viremia, strong immune responses, severe dengue symptoms, and even lethal infections observed in these immunocompromised mice, these models have several limitations: (a) highly reliant on adapted strains; (b) mice perish from central nervous system infection; (c) absence of natural immune responses thereby impacting its suitability for vaccine research; (d) cytokine storms under IFN signalling suppression.

#### Humanized mouse model for DENV

Humanized mice, as immunodeficient murine models engrafted with human cells or tissues, provide a sophisticated platform for the integration of diverse human components and are indispensable in investigating infection or disease processes in humans (Table 5). The investigations of humanized mice have focused on DENV-2 (Table 5). DENV infected human peripheral blood lymphocytes to reconstitute SCID (SCID-HuPBL) mice has a notably low infection efficiency [66]. NOG/SCID mice have the potential to replicate mosquito-borne transmission and clinical manifestations of DF, and to assess anti-viral compounds [67]. NOD-SCID IL2rγ mice (NSG) can be employed to investigate the role of cross-reactive T cells in DENV primary and secondary infections [68,69]. NOD/SCID-human CD34^+^ mice can assess the virulence potential of various clinical isolates of DENV, whereas did not manifest DHF/DSS [67,70,71]. Humanized mouse models NSG, NOD/SCID, RAG, and NRG mice all lack specific immune cell lineages and functional humoral immune responses. NSG-BLT mice promise in DENV vaccine evaluation and secondary infections through IgM and nAbs induction and T cell responses [72]. NOD/SCID-BLT mice can be used for drug and pathogenesis research, but not for vaccine research [73]. NSG transgenic mice expressing human HLA I class (HLA-A2), called NSG-A2, are applied to assess CD4+ and CD8+ T cell responses [74]. NSG-SGM3-BLT mice have higher antigen-specific IgM and IgG levels after DENV infection compared with NSG-BLT mice, and can be used for the study of human antigen-specific B cell responses [75]. NRG mice, also known as NOD-Rag1^-/-^IL2rγ^-/-^ double KO mice, contributes to NK cell function investigation of DENV infection [76]. Additionally, IL-15 and Flt3L can optimize NSG mice to produce human NK cells [68]. NRG mice (human HLA-DR4 overexpression) can establish specific humoral immune responses and allow for class switching [77]. Additionally, RAG2^-/-^γc^-/-^ (RAG2 -hu) and RAG2^-/-^γc^-/-^ human CD34^+^ have been tested for dengue fever immune pathogenesis and antibody neutralizing activity [78,79].

**Table 5.** Humanized mice model of Dengue virus infection.

Mouse model	Serotype	Clinical signs	Primary application	Limitations	Refs
SCID-HuPBL	DENV-1	Low blood viremia	Dengue pathogenesis	Low infection efficiency; Lack humoral and cellular response	[66]
NOD/SCID IL2rγ (NSG)	DENV-2	Viremia, fever, liver damage, thrombocytopenia	Dengue pathogenesis; Antiviral compounds	No signs of DHF/DSS; DENV-specific T cell responses	[68,71]
NOD/SCID-BLT	DENV-2	Viremia, fever, thrombocytopenia, elevated cytokines, IgM antibody	Pathogenesis mechanism; Antiviral drugs	No signs of severe dengue; Lack humoral response; ADE	[73]
NOD/SCID-human CD34+	DENV-2	Fever, thrombocytopenia, viremia, DENV detected in organs	Antiviral compounds	Lack both humoral and cellular response	[67,70,71]
NSG-A2	DENV-2	Viremia, T-cell response, elevated cytokines	Immunogenicity; T-cell immune response	Lack humoral response	[74]
NSG-BLT	DENV-2	Viremia, fever, thrombocytopenia, specific IgM antibody	Immunogenicity; Subsequent DENV infections	Lack humoral response; ADE	[72]
NSG-SGM3	DENV-2	High viremia, fever, immune response, thrombocytopenia, plasma IgM and IgG	B cell development and response; Cytokines production	Lack cellular response	[75]
NRG	DENV-2	High blood viremia, fever, thrombocytopenia, cytokine	Immune response; Hematolymphoid system	Lack of NK cells	[76,77]
RAG2^−/−^γc^−/−^ (RAG-hu)	DENV-2	Viremia, fever, antibody production	Immunopathogenesis; Potential vaccine development	Natural immune	[43,79]
RAG2^-/-^γc^-/-^human CD34+	DENV-2	Viremia, thrombocytopenia, fever, elevated cytokines, nAbs against DENV, IgM and IgG	Virulence evaluation; Potential antibody responses against DENV	No signs of DHF/DSS; No functional antiviral immune response	[43,79]

NSG mice expressing human stem cell factor commercially named NSG-SGM3.

**Table 6.** Animal model for ADE.

Mouse model	Serotype	Advantages	Disadvantages	Refs
Rhesus monkey	DENV2/4	Physiological and immunological responses; Higher titres and prolonged viremia under ADE;	Lack clinical signs;	[31,85]
Marmoset	DENV1/2/3	Prolonged viremia in secondary infection;	Lack clinical signs;	[34]
NOD/SCID-human CD34+	DENV-2	ADE and cross-reactive T-cell activation	Costly and time-consuming; Animal-to-animal variation; Lack severe manifestations	[44,67]
AG129	DENV2/3/4	Viremia, vascular leakage, DHF/DSS under hetero-serotype infection; poorly nAbs; mortality;	Immune deficiency; Unavailable direct production of nAbs; ADE-independent lethal diseases	[44,86]
LysM-Cre ^+ ^IFNAR^fl/fl^	DENV2/3	neutralizing cross-reactive response with DENV2/3, severe plasma leakage, hypercytokinemia, liver injury, hemoconcentration, and thrombocytopenia	Further identification of ADE	[61]

In summary, despite the inherent limitations of humanized mouse models, such as high costs and functional deficiencies in T and B cells leading to impaired humoral and cellular immunity, they still serve as a valuable tool for studying the pathogenesis of human dengue fever [43]. These models exhibit symptoms such as fever, viremia, erythema, and thrombocytopenia without reported severe cases. Additionally, they provide additional evidence for innate and adaptive immune responses to dengue infection and play a critical role in drug development. Nevertheless, overcoming challenges associated with the engraftment of human stem cells and lymphocytes, such as extended time requirements, interspecies variations, xenograft-related host diseases, donor-recipient heterogeneity issues, and the comprehensive reconstruction of immune system components is imperative.

### Swine model

Yucatan miniature pigs detect viremia with DENV-1 subcutaneous injection [11], but not following intravenous injection [11,80]. Further studies are warranted to investigate the susceptibility, viremia, clinical manifestations, and immune response other dengue virus serotypes (Table 2).

### Tree shrew model

The tree shrews, belonging to the family *Dryopsidae* and the genus *Dryopithecus*, exhibit a higher genetic similarity to primates than to rodents [11,81]. They have been found susceptible to all four DENV serotypes, with very low viremia and intracerebral pathological changes following DENV-2/3 [11,82]. However, their utilization is currently limited due to the absence of specific reagents and inbred lines for this species [11,82].

## The ADE in animal model – far from the real infection world

Antibody-dependent enhancement is the phenomenon concerned widely, in which virus-specific antibodies, typically non-neutralizing antibodies, bind to the viruses, and the virus-antibody complexes then bind to FcRs on certain cell surface or complement, subsequently enter those cells, thereby mediating virus infection of these cells and enhancing the infectivity of the virus [16]. Studying the ADE phenomenon serves to enhance our understanding of dengue fever’s pathogenesis, providing a scientific foundation for devising more efficacious treatment strategies regarding clinical treatment (Table 6). Additionally, early intervention can mitigate the risk of ADE occurrence and aid in accurate diagnosis and targeted treatment planning, thereby reducing patient mortality rates. Moreover, immune modulators may be employed to attenuate antibody enhancement against the virus. In terms of vaccine development, investigating the ADE phenomenon contributes to crafting safer and more potent vaccines against dengue virus, diminishing vaccine-induced ADE risks while bolstering vaccine protective efficacy.

Primary dengue infection provides lifelong immune protection against the primary dengue serotype; however, heterogeneous infection of DENV not only fails to provide immune protection but also increases the likelihood of severe DF [83,84]. Many studies have attributed severe DF to ADE, according to the standard on higher viremia and more severe clinical manifestations [83,84]. To address ADE phenomenon, such models possessed at least three characteristics are urgently needed: susceptible to all four serotypes, normal immune with nAbs production and induction of a robust and balanced ADE immune response. The positive progress has been achieved upon the animal models investigation of ADE. The first documentation of DENV induced ADE-like phenomena has been reported in young macaques, resulting in a 3-100-fold increase in viremia [31,85]. Furthermore, vaccinated macaques exhibited elevated viremia and cytokines [30]. When pre-existing antibodies are present, AG129 pups infected with DENV-2 exhibit ADE phenomenon, ultimately leading to early mortality [86]. Additionally, neutralizing cross-reactive anti-DENV monoclonal antibodies post-infection with DENV-2/3 in LysM Cre^+^ IFNAR^fl/fl^ mice resulted in ADE [61]. Currently there are less humanized mouse models that replicate ADE phenomena *in vivo*. NOD/SCID-human CD34^+^ may eventually also prove useful for understanding ADE of infection and cross-reactive T-cell activation [67]. RAG2^-/-^γc^-/-^ human CD34^+^, NSG-A2 and NSG-BLT models are potentially valuable for investigating the mechanisms underlying dengue-induced ADE [74,79,87].

In conclusion, NHP models fail to replicate the ADE phenomenon observed in severe and fatal dengue hemorrhagic fever in humans. Similarly, the ADE phenomenon induced by natural infection cannot be replicated in immunodeficient murine animal models.

## No animal model can reproduce cytokine storms of human beings

Dengue viruses infect primary target cells, such as monocytes, macrophages, dendritic cells, and immature CD4+ and CD8+ T cells. These cells produce significant amounts of acute phase proteins, cytokines, and chemokines including CCL2 (MCP-1), CCL3 (MIP-1α), CCL4 (MIP-1β), and CCL5 (RANTES) et al [4,88]. Severe dengue fever, secondary infections, and ADE phenomena are intricately linked to dysregulated cytokine responses [89,90]. Based on the cytokine profiles, they mainly can be classified into Th1 and Th2 subtypes. While Th1 cells secrete IFN-r,TNF-a and IL-2,Th2 cells produce a range of interleukins including IL-4/5/6/10/13 [90,91]. Most studies have reported the secretion of both Th1 and Th2 cytokines by DENV-specific T cells; however, there is still ongoing debate regarding their respective predominance. Monocytes demonstrate an increase in IL-10 following infection with DENV-2 (strain16681), particularly through the ADE pathway [92]. This corresponds to elevated levels of IL-10 and reduced IFN levels in severe dengue patients (DENV-2/3) [90,93–95], suggesting a close association between Th2-type cytokines and the severity of dengue virus infection. An inclination towards a Th1 response (IFN-γ/IL-4 ratio) was observed in the immune reaction to DENV 2 in C57BL/6 mice compared to BALB/c mice [45]. Furthermore, infection with DENV-1 in adult C57BL/6 mice resulted in an increase of the pro-Th1 cytokines IL-12 and IL-18 [96]. The compromised IFN response in AG 129 mice restricts their suitability for accurate evaluation of the impact of dengue infection on the balance between Th1/Th2 responses. In AG129 mice, infection with DENV-1 leads to increased IL-1α/6/10/12 (p40)/12 (p70), IFN-γ, and G-CSF [59]; DENV-2 infection results in elevated IFN-γ, IL-6, and TNF-α [55]; DENV-3 infection induces upregulation of TNF-a, IFN-γ, IL-3/4/6/10/12p40/12p70, CCL2/3/4/5 and G-CSF [58,59]; while DENV-4 infection causes an increase in TNF-α, IFN – γ, IL – 1 α/6/10/12(p40), CXCL1, CCL2/3//4//5 and G-CSF [97]. In CCR1^-/-^, CCR2^-/-^ and CCR4^-/-^ mice with DENV-2 infection, both serum IL-6 and IFN-γ are elevated [98]. It is noteworthy that cytokines such as IL-13/17/21 have also been implicated in the pathogenesis of DENV-2, warranting further investigation. Hence, the cytokine storm triggered by dengue infection is a multifaceted process that is modulated by variables such as the genetic background of the animal model, viral serotype, immune status and clinical manifestations. In severe dengue infections in humans, IL-10 levels are elevated while IFN levels are decreased; however, current animal models show elevated levels of both IL-10 and IFN. Ultimately, it is evident that no existing animal model can faithfully replicate cytokine storms in humans.

## Conclusions and perspective for future animal model

This year has witnessed a surge in dengue virus cases, particularly in Indonesia and Brazil. To effectively manage the dengue epidemic, it is imperative to establish a robust animal model. Currently, no animal models can closely recapitulate clinical manifestations of human to all dengue virus serotypes for preclinical testing studies. Moreover, the clinical responses of available animal models vary depending on the host category, immune status, serotype, variant strain, vaccinated dose, inoculation route and frequency of infection. Consequently, understanding the susceptibility of animal models to all four serotypes of dengue virus and newly evolved viral strains resulting from selection pressure, as well as comprehending the clinical manifestations of DHF/DSS, presents a significant challenge for future development of dengue animal models. Furthermore, addressing ADE phenomena induced by sequential infection with heterotypic dengue viruses and by vaccination, DENV receptors in vivo, and cytokine storms are paramount priorities for advancing research on dengue animal models and evaluating dengue vaccines. Therefore, creating an immunocompetent animal model possessing specific receptors susceptible to all four serotypes of DENV with robust ADE responses could significantly accelerate efficiency towards vaccine development. Furthermore, such models should accurately simulate systemic bleeding, organ failure and immune storm as seen in human DENV infections.
